# Nature-Inspired Micro/Nano-Structured Antibacterial Surfaces

**DOI:** 10.3390/molecules29091906

**Published:** 2024-04-23

**Authors:** E Jin, Zhijun Lv, Yinghao Zhu, Hongmei Zhang, He Li

**Affiliations:** School of Mechanical and Electrical Engineering, Henan Agricultural University, Zhengzhou 450002, China; jine2018@163.com (E.J.); lvzhijun@henau.edu.cn (Z.L.); zyhzyh@henau.edu.cn (Y.Z.); zhanghongmei0905@henau.edu.cn (H.Z.)

**Keywords:** bioinspired, micro-/nano-structured, biomimetic, antibacterial surfaces

## Abstract

The problem of bacterial resistance has become more and more common with improvements in health care. Worryingly, the misuse of antibiotics leads to an increase in bacterial multidrug resistance and the development of new antibiotics has virtually stalled. These challenges have prompted the need to combat bacterial infections with the use of radically different approaches. Taking lessons from the exciting properties of micro-/nano-natural-patterned surfaces, which can destroy cellular integrity, the construction of artificial surfaces to mimic natural functions provides new opportunities for the innovation and development of biomedicine. Due to the diversity of natural surfaces, functional surfaces inspired by natural surfaces have a wide range of applications in healthcare. Nature-inspired surface structures have emerged as an effective and durable strategy to prevent bacterial infection, opening a new way to alleviate the problem of bacterial drug resistance. The present situation of bactericidal and antifouling surfaces with natural and biomimetic micro-/nano-structures is briefly reviewed. In addition, these innovative nature-inspired methods are used to manufacture a variety of artificial surfaces to achieve extraordinary antibacterial properties. In particular, the physical antibacterial effect of nature-inspired surfaces and the functional mechanisms of chemical groups, small molecules, and ions are discussed, as well as the wide current and future applications of artificial biomimetic micro-/nano-surfaces. Current challenges and future development directions are also discussed at the end. In the future, controlling the use of micro-/nano-structures and their subsequent functions will lead to biomimetic surfaces offering great potential applications in biomedicine.

## 1. Introduction

So far, persistent bacterial infections and contamination have occurred widely and caused a large number of problems in various sectors, including the clinical medicine and food industries, water purification systems, sewage treatment plants, bioleaching, industrial aquaculture, and other industries [1,2,3]. On Earth, over 99% of bacteria are thought to live in structured biofilm communities [4]. Generally, bacteria mainly exist in the form of biofilms [5], and device- and non-device-associated infections are associated with bacterial biofilms. Data for device-related infections have been estimated as follows: 2% of breast implants, 2% of joint prostheses, 4% of mechanical heart valves, 10% of ventricular shunts, 4% of pacemakers and defibrillators, and about 40% of ventricular-assisted devices [6]. Biofilms that are formed by drug-resistant *Pseudomonas aeruginosa* are a leading cause of death in hospital burn centers worldwide [7]. Highly resistant biofilms can form in the host, leading to chronic and persistent disease. The National Institutes of Health (NIH) revealed that among all microbial and chronic infections, 65% and 80%, respectively, are associated with biofilm formation [8]. For example, dental caries, sinusitis, otitis media, osteomyelitis, and endocarditis, especially nosocomial infection, are all related to the formation of pathogenic bacteria biofilm [9,10]. *Pseudomonas aerobicus* and *Fusobacterium nucleatum* are one of the causative agents of periodontitis. These microbes also have the ability to form biofilms on a variety of surfaces, including mucosal surfaces in the oral cavity. In recent years, various medical devices have been widely developed, such as catheters, pacemakers, artificial valves, orthopedic implants, etc. However, device-associated infections have become a serious problem in clinical practice and the National Institutes of Health believes that more than 75% of tibial micro-infections are caused by bacterial biofilm within the human body. The failure of medical implant devices and 60–70% of nosocomial infections are closely related to biofilm formation [11]. Therefore, the development of therapeutic measures that can reduce bacterial infection has gradually become the focus of scientific and medical research. The initial research focused on methods of adding antibacterial or antimicrobial agents, including nanoparticles, antibiotics, and polymers. Due to several significant disadvantages, such as causing tissue sensitivity toxicity and increased antibiotic resistance in patients, as well as concerns about environmental toxicity and dosage complications, additive approaches have become less attractive as a long-term antimicrobial solution [12].

As a result, some studies have explored alternative ways of killing bacteria through the physical contact mechanism rather than antibiotics. These developments were partly inspired by nature. Bionics refers to the science of human inventions by mimicking biological functions, and it is a new frontier discipline. The research object is the structure, function, and working principle of the organism, and these principles are transplanted into artificial engineering technology. The advent of this discipline has greatly broadened the technical horizon of human beings, showed huge development potential, and is the crystallization of human wisdom. There are countless examples of bionics: bats and radar, eggshells and thin-shell architecture, sharks and drag reduction materials, bees and bionics, dragonflies and bionics, and so on. In this review, we use the abundance of functional surfaces provided by nature as inspiration models for synthetic paradigms, because these properties are unmatched by today’s artificial materials and are a direct result of evolutionary pressures that force natural species to become highly optimized and efficient. Bionic materials provide innovative solutions for the design of a new generation of functional materials and can lead to new material design principles. At present, a large number of biomimetic functional materials are involved in the research field of water-repellent, self-cleaning, drag and friction reduction in fluid flow, energy conversion and conservation, adhesion, antifouling, antibacterial, and self-healing properties. All these special features are demonstrated by natural systems and cleverly designed structural features based on a wide variety of biological surfaces through a complex control of length scales. Thus, natural surfaces are organized in a rather complex way, showing a hierarchical structure on all length scales. The current situation of bactericidal and antifouling surfaces with naturally occurring and bionic micro-/nano-structures is reviewed. The bionic surfaces achieve extraordinary biological activity, and antifouling, antibacterial, and many other applications. At the same time, the antibacterial mechanism behind the function demonstrated by the natural surface prototype will be analyzed and discussed. In addition to showing the potential and significance of biomimetic surface structures based on chemical groups, small molecules, and ion functionalization, it will also describe existing limitations and discuss emerging possibilities and prospects.

## 2. Learning from Nature: Design Principles and Antibacterial Applications

**Figure 1:**
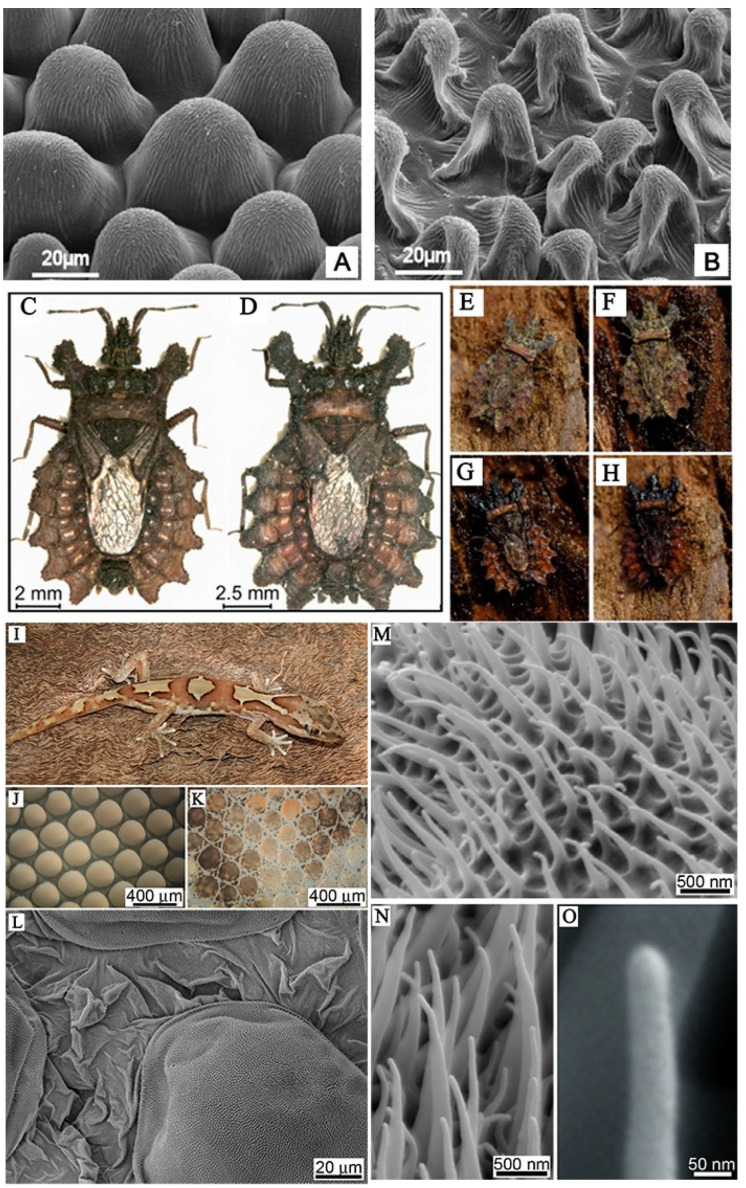
(**A**,**B**) Scanning electron microscope (SEM) micrographs of the upper (frontal) surface of *Dahlia* flower leaf (petal) under different treatments [13]. (**C**,**D**) Bark bugs of different sizes, (**E**) camouflage of dried animals on dried bark; (**F**) camouflage of dry animals on wet bark; (**G**) camouflage of wet animals on wet bark; (**H**) camouflage of wet animals on dry bark [15]. (**I**) Photograph of the gecko *Lucasium steindachneri*; (**J**) the microstructure of the outer skin of the gecko abdomen and (**K**) the microstructure of the dorsal area of the gecko; (**L**) topographical SEM image of the epidermal dome regions and areas between scales on the dorsal region of the lizard; (**M**–**O**) micro-/nano-structure of dorsal scales of gecko [17].

Due to natural selection and evolutionary pressures, species have developed a variety of submicron surface morphologies and structures. These naturally occurring surfaces have also developed their own protective mechanisms to cope with pathogen infections, which play a critical role in the functional adaptability of environmental conditions. In many instances, surface properties must be preserved to maintain their survival function, as shown in Figure 1. For example, the plant’s outermost protective layer is a continuous extracellular membrane called the stratum corneum. The plant cuticle can overcome many physical and physiological problems, such as the dryness of the surrounding environment; self-cleaning performance ensures that plants can overcome the infection of diseases and pests; special optical properties protect it from harmful radiation; resistance of mechanical properties to mechanical stress, and so on [13]. Gorbet et al. selected the garden dahlia plant *Dahlia pinnata* and the hovering fly *Eristalis tenax*, examined leaves, petals, and flower stems using cryo scanning electron microscopy, and performed force measurements of fly attachment to surfaces of these plant organs. The results showed that insect attachment on the petal surface was significantly weaker compared to that on smooth leaf and smooth reference glass. The papillary epidermal cells on the petals have micro- and nano-scale keratin folds, which is the main reason for reducing insect attachments [14]. Researchers have found that by virtue of their excellent camouflage, flatbugs can hide almost invisibly in tree bark. The bark darkens when wet by rain, and flatbugs change their optical properties accordingly. This fascinating camouflage is due to the presence of the chemical erucamide in the hydrophilic cuticle surface wax layer, which is enhanced by a large number of columnar surface microstructures [15,16]. For gecko skin, attention has been paid to its unique micro-/nano-structure, which not only acts as a self-cleaning feature, but also provides a superhydrophobic, anti-wetting, and antibacterial barrier as a potential natural template for the artificial application of specific controls [17]. Li et al. showed that gecko microspinules (hairs) and their equivalent replicas, bearing nano-scale tips, can kill or impair surface-associating oral pathogenic bacteria with high efficiency even after 7 days of repeated attacks. Scanning Electron Microscopy suggests that there is more than one mechanism contributing to cell death, which appears to be related to the scaling of the bacteria type with the hair arrays and accessibility to the underlying nano-topography of the hierarchical surfaces [18]. In conclusion, nature-inspired structures can influence our design principles for a variety of future applications.

### 2.1. Antifouling

In most cases, in order to avoid or reduce unnecessary biological fouling in the environment that affects surface properties and functions, various biological species can solve this problem through complex surface micro-/nano-structures. These natural surfaces take advantage of their microscopic and nano-structural characteristics, which are arranged on different terranes, endowing them with biological properties. Previous studies have shown that plants and animals have developed antifouling surface topologies that can reduce biofouling, thereby controlling the development of bacteria and their biofilms. The micro-/nano-structures on the surface of the lotus leaf are a prominent representative; Liang et al. established a lotus leaf-like surface on the surface of a porous substrate and revealed its antifouling performance. Firstly, the complex nano-/micro-structures and low surface energy of the superhydrophobic surface hinder protein adhesion and bacterial adhesion on the surface. Secondly, the sharp surfaces constructed by the nanofibers disrupt bacterial cell integrity, providing physical antibacterial action. Thirdly, the coating releases silver ions and reactive oxygen species, which are produced by silver nanoparticles, to chemically damage bacteria. Through the synergy of superhydrophobic surface and silver nanoparticles, the coating achieves good resistance to bacterial adhesion and is able to eliminate *Escherichia coli* (100.0 ± 0.1%) and *Staphylococcus aureus* (99.8 ± 0.1%) [19]. Lv et al. reported the preparation and oil–water separation performance of modified copper foam bioinspired by lotus leaf’s micropapillae structure. In the preparation process, the micropapillae structure similar to lotus leaf was obtained on the surface of foam copper. The hierarchical structure endowed copper foam surface roughness and low energy, which were indispensable for superhydrophobic characteristics. The modified copper foam also exhibited simultaneous superhydrophobic (water CA was 158.2°) and superoleophilic (oil CA was 0°) features, and separation efficiency could reach above 95% for various oily mixtures [20]. It has been found that hydrophobic surfaces containing micro-/nano-structures exhibit superhydrophobicity. In the case of lotus plants, the perfect condition for self-cleaning must be a combination of surface chemistry and a specific structure that leads to a significant reduction in the contact area of water droplets. Since these anti-biofouling terrains do not require action against microbial targets, this strategy does not promote the development of resistance associated with chemical methods [20,21,22,23,24,25]. The nano-scale topology on the wings of cicada and dragonfly also has antibacterial activity, which can be used as a bionic prototype to control biological deposition [26,27]. In addition, marine animals have adopted a similar antifouling strategy by using surface micro-/nano-structures to improve their survival. The combination of the surface topography of shark’s skin and mucosal coating is the main reason for the antifouling properties of sharks and other fish [28,29]. Live soft coral molts in unfriendly environments, and the secreted mucus defends itself against fouling microorganisms, as mucus contains a wide variety of toxic components, which have bactericidal effects [30]. Basic information on different organisms inspiring scholars is shown in Table 1.

### 2.2. Antibacterial

In terms of the biological significance of the antifouling of lotus, the micro-/nano-structures of leaf surfaces provide significant self-cleaning and antifouling surface properties, and self-cleaning plays an important role in preventing a pathogen invasion of the leaf surface. Many fungal spores and bacteria require water to germinate and can infect leaves in the presence of water. Therefore, removing water can minimize the chance of infection. In addition, removing dust particles from leaf surfaces can minimize changes in plants, such as overheating or salt damage. Although lotus leaves have been used as hydrophobic and self-cleaning model surfaces, many other biological surfaces exhibit similar properties [19,35]. Most natural antibacterial surfaces have evolved to possess a high-aspect-ratio nano-/micro-structure morphology to protect them from pathogenic infestation. Such as rose petals, which possess hierarchical structures like the micro-papillae measuring tens of microns and nano-folds that range in the size of 700 ± 100 nm. By testing the efficacy of these artificial surfaces in preventing biofilm growth using clinically relevant bacteria strains, even after prolonged growth (several days), the biomass of *Staphylococcus epidermidis* (63.2 ± 9.4% less) and *Pseudomonas aeruginosa* (76.0 ± 10.0% less) biofilms were significantly reduced on rose-petal-structured surfaces, in comparison to flat surfaces. Demonstrating that hierarchical structures are more effective in delaying biofilm growth by comparing *Pseudomonas aeruginosa* growth on representative unitary nanopillars [36]. A common feature of these surfaces is that their special wettability is a direct result of the chemical synergy between the morphology of the micro-/nano-structure morphology and hydrophobic surface chemistry. To avoid bacterial infection, when the wings of dragonfly and cicada contact bacteria, the morphology of nano-structures on their surface can penetrate the cell membrane, resulting in bacterial rupture [25,37]. Notably, this strategy avoids the development of bacterial resistance to traditional antibiotics because of the physical bactericidal properties of nano-structures [38]. In research on the great potential of bactericidal properties of micro-/nano-structures, theoretical calculations have been carried out for in-depth analysis in order to better understand the mechanism and apply biomimetic methods to artificial surfaces.

## 3. Bionic Artificial Micro-/Nano-Structures and Antibacterial Mechanisms

In hospitals and high-risk environments, such as medical equipment doorknobs, bedside tables, bed rails, and other high-risk surfaces, the design of surfaces that can inhibit the adhesion and proliferation of bacterial pathogens is critical to minimize the spread of multidrug-resistant bacteria. In order to be more economical and easily applied to existing high-risk surfaces and prevent the spread of pathogens through surface contamination, a large number of antibacterial and antifouling coatings need to be developed urgently. However, it is interesting to note that the micro-/nano-morphology of the surface is a feasible strategy for controlling bacterial adhesion. This Section provides an overview of common nano-structures and antibacterial mechanisms.

### 3.1. Types of Micro-/Nano-Structures

The common feature of antibacterial or antifouling surfaces is the existence of a continuous array of micro-/nano-structures with different morphologies. Micro-/nano-structures destroy the bacterial cell wall when they adhere to the surface by a physical killing mechanism. Therefore, these interesting structures have inspired scientists to create a series of artificial bactericidal surfaces with bionic micro-/nano-structures [37]. Importantly, these interesting surface properties can be transformed into synthetic biomimetic surfaces when simulating natural structures, resulting in a significant increase in the biological properties of such surfaces. For example, in order to mimic the dense nano-columnar structures that cover cicada wings, Yuan et al. developed a universal and simple method to grow biological nano-daggers on a variety of surfaces, giving them high bactericidal efficiency and selectivity [39]. Sabra et al. designed a surface that interfered with the formation time of *Staphylococcus aureus* biofilm based on the morphology of shark skin [28]. Hochbaum et al. found that when bacteria interact with ordered nano-scale columns, they can induce bacterial sequencing and directional attachment [40]. Xu et al. demonstrated that microtopographic surface patterns of spatial organization are a promising method for controlling or inhibiting bacterial adhesion and preventing further biofilm formation [41]. Kargar et al. used single polystyrene nanoparticles to produce continuous and tightly packed particle layers on the surface for bacterial inhibition studies. They observed that the layer of colloidal particles with a size of 630–1550 nm effectively reduced bacterial attachment and biofilm formation [42]. Pingle et al. used self-assembled spherical micron silica and nano-polymethylmethacrylate-sized particles to make a topographic model of binary colloidal crystal (BCC) to study bacterial adhesion; SEM images of the BCC assembly surfaces showed that the 3 to 5 µm-sized pattern could easily trap the bacteria and potentially delay bacterial biofilm formation [43]. The results show that the model bacteria have the ability to be selectively arranged and controlled by topological surfaces, showing that binary-particle-assembled microtopography is a promising method to prevent the initial adhesion of bacteria on different substrate surfaces. The bacteria-killing model inspired by nature provides us with a new way of using antibacterial and antifouling biological materials. Natural surfaces cannot be arbitrarily customized, designed, or optimized because they are the result of natural selection. However, once the structural characteristics of these surfaces are understood, they can be optimized through the development of biomimetic artificial micro-/nano-simulators.

**Figure 2:**
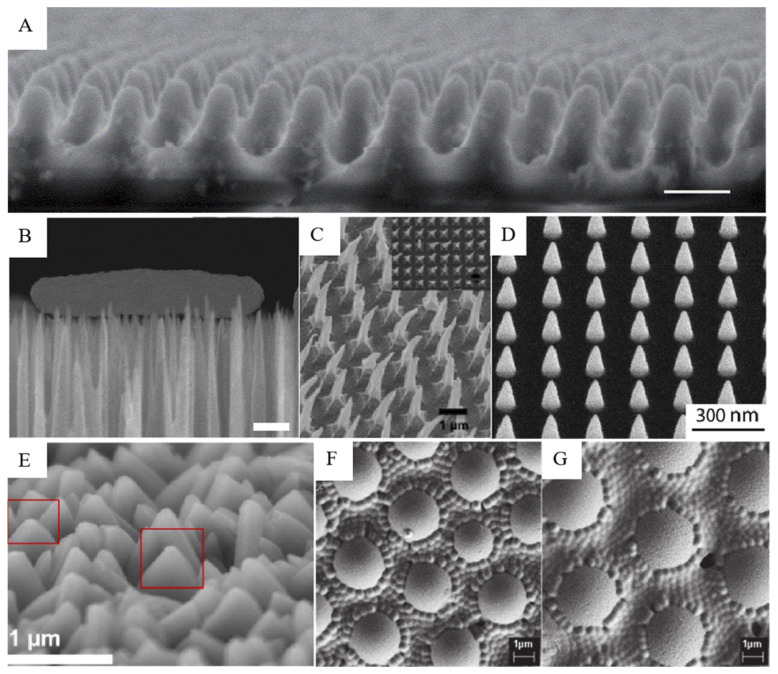
Bionic micro-/nano-structures on the surface of the analog. SEM images: (**A**) moth-eye imaging of simulated terrain [45]; (**B**) *Escherichia coli* interacts with the black silicon surface, scale bars 500 nm [46]; (**C**) hooked polystyrene films based on three-dimensional nanopyramids [48]; (**D**) cone-shaped nano-structures fabricated using electron beam induced deposition technology [49]; (**E**) dagger-like structure of zeolitic imidazolate framework coatings on glass (circled in red box) [39]; (**F**,**G**) spherical and hemispheric allylamine plasma polymer coatings on glass [43].

There are many types of micro-/nano-structures synthesized by biomimetic methods that have been used for their different biological properties. Bionic micro-/nano-structures synthesized on the surface of simulants can be roughly divided into seven types: columnar [44,45], needle [46,47], hook [48], cone [49], spherical/hemispheric protrusion [42,43], and others [41]. Depending on the manufacturing technology, different forms of micro-/nano-structures can be manufactured, including different size, density, depth, stiffness, and surface chemistry, as shown in Figure 2.

### 3.2. Antibacterial Mechanism of Micro-/Nano-Structures

**Figure 3:**
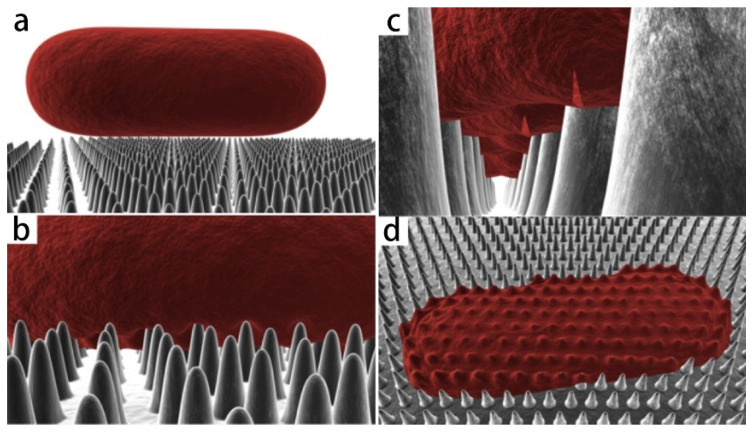
Three-dimensional schematic diagram of simulated interaction between rod-shaped bacteria model and cicada wing. As the cell comes into contact (**a**) and adsorbs onto the nanopillars (**b**), the outer layer begins to rupture in the regions between the pillars (**c**) and collapses onto the surface (**d**) [26].

In-depth studies of naturally occurring micro-/nano-structured surfaces have led to novel bactericidal paradigms that work by a range of physical antibacterial surfaces. A physical antibacterial mechanism is a method to kill or remove pathogenic microorganisms by physical factors. Researchers have shown that when the micro-structural pattern size on the surface is close to the size of the bacteria, the contact points provided for the bacteria will be reduced, resulting in a repellent effect on bacteria [50]. The elimination of microbes on the surface was actualized by the physical rupture of the microbe cell wall by nanoprotusions, without any involvement of chemical species [51]. In general, the physical sterilization mechanism includes three actions: (1) the internalization or insertion of some particles in the micro-/nano-structure, resulting in functional damage of the membrane; (2) contact puncture mechanism; and (3) non-contact mechanical deformation mechanism. The adhesion and interaction between micro-/nano-structures and bacteria is the key to physical killing. Here, we discuss the physical factors of the contact killing mechanism between the micro-/nano-structures and bacteria. For example, studies have shown that adhesion can be transformed into bending stress and stretching forces on bacterial membranes. The biochemical properties of the surface can regulate the strength of adhesion [52]. On the nano-structured surfaces, due to the increase in contact area, the adhesion results in higher tensile and bending values compared to flat surfaces. For example, Pogodin et al. found that membranes are more likely to rupture when bacteria attach to areas of high tensile force between pillars, as exhibited in Figure 3 [29]. Bactericidal properties of nano-structured surfaces are strongly dependent on surface roughness, for example, higher surface roughness results in higher bending and tensile forces. The antibacterial surface structures of dragonflies and cicadas are at least 10 times larger than bacterial membranes [53]. Different bacterial membranes have different tensile moduli, and they can withstand the corresponding tensile and bending forces. Therefore, bacterial membranes with lower tensile modulus are more likely to rupture on nano-structured surfaces [29]. However, for the non-contact mechanical deformation mechanism, early studies have shown that bacterial membranes also exhibit different mechanical properties with changes in environmental conditions [54]. Some researchers have proposed a mechanical theoretical model in which the mechanical deformation of bacterial membranes is the result of the tensile forces generated on the nano-structures during the pursuit of stable attachment [45]. In addition, the interaction strength between bacteria and substrate depends on the magnitude of the force. Gravity, van der Waals repulsion, and hydrophobic interactions generally exist between the bacterial membrane and the surface of the array [55]. Xie et al. found that gravity may induce cell membrane penetration when the radius of the nanowire is less than 10 nm. For nanowires with a radius of 50 nm, the force required to penetrate the membrane is on the order of nN, one order of magnitude higher than its gravity [56]. In terms of mechano-bactericidal micro-/nanomaterials, various studies have reported that surface charges have been used to fight bacterial infection, in which electrostatic attraction plays a key role. There are a large number of anionic phosphate head groups on the membrane of Gram-positive bacteria and Gram-negative bacteria. These negatively charged head groups have strong electrostatic interaction with the modified positively charged ZIF-L (zeolitic imidazolate frameworks) nano-dagger array, resulting in bacterial contact puncture death, which has high sterilization efficiency and selectivity [39]. In addition, hydrophobicity is also considered a bioactive and antibacterial modification property. The self-cleaning properties of such surfaces are activated by gas traps formed in the liquid of superhydrophobic surfaces in some plants [57]. For hydrophobic surfaces, a strong correlation between roughness ratio and bacterial adhesion was obtained, while autocorrelation length (related to the interasperity spacing) was not found to be correlated with bacterial adhesion. For superhydrophobic surfaces, the combination of factors included (i) the surpassing of Laplace pressure force of interstitial air over bacterial adhesive force, (ii) the reduced effective substrate area for the bacteria wall due to air gaps having direct contact, and (iii) the reduction in the attractive van der Waals force that holds adhering bacteria on the substrate (the energy barrier of bacterial desorption/removal) [58].

**Figure 4:**
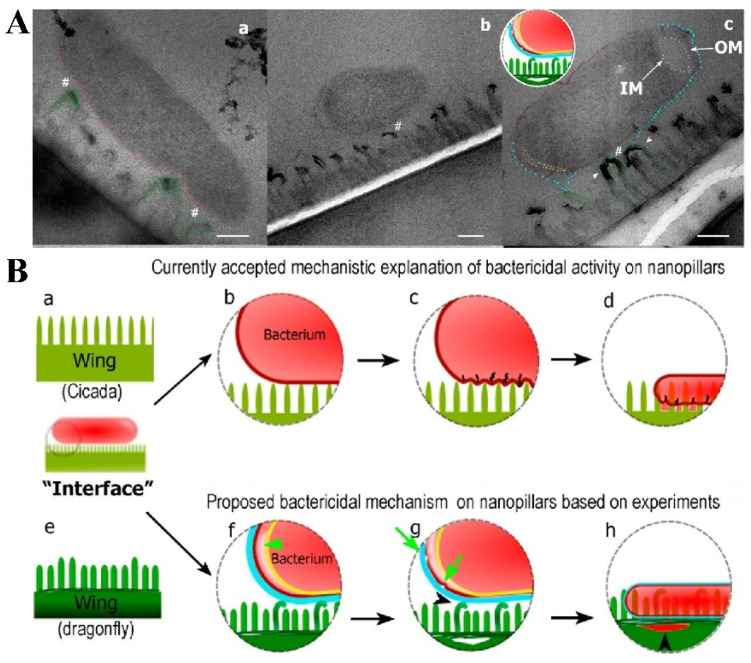
(**A**) TEM micrographs showing bacteria–nanopillar interaction at the interface. (**a**) Longitudinal section of *Escherichia coli* bacterium approached on two tall nanopillars of a dragonfly wing topography. The membrane is still intact and is pressed inward. A nanoscale space between bacterial membrane and nanopillars can be noted (#). This pseudo-nanospace is filled by EPS; therefore bacterium is physically attached to the nanopillars via EPS layer. As EPS does not have sufficient contrast to the surrounding under TEM, we do not see EPS as a separate layer in the image. Bending of the nanopillars underneath the bacterium is highlighted by the green dashed lines. (**b**) Longitudinal cross section showing a separation of the inner membrane (IM) and outer membrane (OM) at the polar ends of the *Escherichia coli* bacterium. The tall nanopillars are bent underneath the bacterium. (**c**) Longitudinal cross section of a bacterium on the dragonfly wing. Bent tall nanopillars are highlighted by white arrow heads. Small gap between nanopillars and the bacterial membrane is still observed (space between red and blue lines, #). Increased membrane separation at the polar end of the bacterium is present. All scale bars correspond to 200 nm. (**B**) Proposed mechanism of bactericidal activity of nanopillars. The mechanism of bactericidal activity based on current accepted mechanistic models using cicada wing structure is shown in (**a**–**d**). The proposed mechanism based on the experimental studies in this work (using dragonfly wing) is shown in (**e**–**h**). (**a**) Cross section of a cicada wing was used for the current studies to determine bactericidal activity. All nanopillars are assumed to be the same in height. (**b**) A bacterium approaches on the surface, and the membrane starts to compress due to weight and adsorption. (**c**) The membrane starts to rupture between attached nanopillars due to stretching. The energy for stretching and membrane deformation is provided by the initial adsorption. (**d**) Once cell membrane ruptures, the bacterium’s cytoplasm leaks, leading to cell death on the nanopillar surface. (**e**) Illustration of the dragonfly wing’s two prominent nanopillar populations. (**f**) Once bacteria approach to the surface, taller nanopillars are being bent by the bacterium. The nanopillars do not puncture the membrane. Bacterium adheres to the nanopillars by the secreted EPS layer and the pilus structures. Once adhesive forces apply stress on bacterial membrane, the two cell membranes of the bacterium start to separate from each other (indicated by the arrow). The EPS layer is displayed in blue, the outer membrane in dark red, and the inner membrane in yellow. (**g**) The damaged bacterial membrane starts wrinkling and forms blebs (arrows), with separation of the nanopillar layer from the wing base, also observable due to the attempts made by the bacterium to move away. (**h**) Once the bacteria die on nanopillars, cytosol is leaked and flows under the nanopillar layer filling the crack formed in the previous step. Nanopillars can be seen inside the bacterium at this stage.

Although many studies believe that the direct interaction between nanopillars and bacteria is the cause of bacterial death, some scientists have put forward different views. Bandara et al. studied the mechanical properties of dragonfly wing nanopillars and the mechanism of interaction between bacteria and nanopillars [59]. They believe that the behavior of bacteria at the nanopillar interface also plays a decisive role in sterilization efficiency. They found that the sterilization mechanism of dragonfly wings was due to the strong adhesion effect between bacteria and nanopillars, and the combined effect of shear force generated by adhesive bacterial movement was important as well. The adhesion of bacteria to the nanopillars is mediated by the bacterially secreted extracellular polymer (EPS), rather than the direct contact between the bacterial cell membrane and the nanopillars because EPS is filled between the nanopillars and the cell membrane (Figure 4A). When the adherent bacteria try to move on the surface of dragonfly wings, the strong van der Waals force generated between the bacterial membrane and the nanopillars causes the membrane to be stretched. And strong shear force will be applied to the bacterial cell membrane when bacteria try to leave the surface of the bactericidal nanopillar interfaces, thus damaging the bacterial cell membrane (Figure 4B). However, the opposite view was also put forward by other researchers. Linklater et al. conducted the same experiment to verify the above results, but they put forward the opposite view. They believe that EPS did not play a role in the mechanical sterilization of nanopillar interfaces [60].

### 3.3. Chemical Effect and Antibacterial Mechanism

In general, the physical effects of the antibacterial effect will cooperate with some traditional chemical damage effects to significantly enhance the antibacterial effect, including as follows: (1) The generation of reactive oxygen species (ROS) destroys the structure of the cell membrane, whereby ROS cuts off the chemical bonds of bacteria’s organic matter to achieve a bactericidal effect [61], thus further damaging nucleic acids, proteins, enzymes, and other cellular components, and causing varying degrees of oxidative stress. Among various nanomaterials, metals and their oxides have been widely studied for their non-toxic, stable, and efficient biological properties [62]. Au, Ag, Zn, ZnO, Fe_2_O_3_, TiO_2_, and other nanomaterials have been widely used in the efficient sterilization of bacteria due to their characteristics of producing ROS. Interestingly, the ZnO nano-column array achieves a stronger bactericidal effect by growing on different substrates. For example, the ZnO nano-column array on zinc surface not only achieves good remote sterilization ability (non-adherent bacteria) by releasing a high concentration of superoxide free radicals, but also has a sterilization effect several orders of magnitude higher than that of the ZnO nano-column array alone. Mujeeb et al. found that silver–copper nanocomposites, synthesized using an Olax scandens leaf extract showed greater antimicrobial activity than monometallic Ag nanoparticles with an increase in ROS production [62,63]. To enable the self-supplying Fenton reaction, iron-containing ferrocene was incorporated into H_2_O_2_-generating polymer micelles to further promote ROS generation, achieving potent killing effects against both *Escherichia coli* and *Pseudomonas aeruginosa* [64]. The aqueous extract of *Phoenix roebelenii palm* leaves has been utilized as an effective chelating/stabilizing agent used to synthesize zinc oxide nanoparticles via a green chemistry approach, exhibiting a significant bactericidal effect towards Gram-positive (*Staphylococcus aureus* and *Streptococcus pneumoniae*) and Gram-negative (*Escherichia coli* and *Salmonella typhi*) pathogenic bacteria [65]. (2) After the contact reaction between metal ions and microorganisms, the release of metal ions will react with microorganisms, resulting in the destruction of inherent components of cells or dysfunction. When a small number of metal ions approaches the bacterial cell membrane, they will be firmly absorbed by Coulomb gravity due to the negative charge of the bacterial cell wall. The metal ions can penetrate the cell wall, and react with a sulfhydryl group (-SH) to coagulate protein composition, damage the activity of cell synthase, and the cell will lose division and reproduction ability, resulting in bacterial death. For example, Gao et al. found that nanobelt arrays combined with the highly effective antibacterial action of Ag showed good antibacterial activity against both Gram-negative and Gram-positive bacteria [66]. More interestingly, Tang et al. reported that combined with the introduction of Au nanoparticles, the photocatalytic effect and mechanical properties of the nano-structures can be significantly enhanced, and the antibacterial effect is very obvious [67].

## 4. Current Challenges and Future Directions

With increasing attention to health care, the development of antibacterial surfaces is also increasing rapidly. The design of antibacterial surfaces requires the ability to repel pathogenic microorganisms, inhibit their attachment and growth, or kill and remove them when they are attached to the surface. However, this design principle has proved difficult. Either the mechanism of inhibiting bacterial attachment and growth cannot fully inhibit the formation of biofilms, or the mechanism of killing bacteria can easily promote the development of bacterial drug resistance. Therefore, it is hoped to achieve good antibacterial effects by constructing unique micro-/nano-structures on the surface of materials. Nature-inspired surfaces, such as insect wings, have the ability to kill cells once attached to them, providing an excellent prospect and template for the development of antimicrobial surface design. The surface contains tunable micro-/nano-arrays that can mechanically destroy the pathogenic cells attached to the surface and kill microorganisms. The precise geometry of the surface topography varies with the substrate, so the properties of these nano-structures ultimately determine the degree of antibacterial effect, adhesion behavior, and biocidal selectivity to specific microbial species. Unlike chemical methods that prevent bacterial attachment, microorganisms cannot easily develop resistance to this method because of the physical mechanism of the biocidal mechanism. This is particularly relevant to the development of biomedical devices, in which pathogenic biofilms can be formed on the substrate surface before or during surgery. Previous research on the bactericidal performance of the bionic surface verified the feasibility of the antibacterial model, which can effectively control the adhesion of bacteria and the formation of biofilm. While nature-inspired micro-/nano-structures have been simulated on a variety of substrates using different techniques, key challenges remain unaddressed, such as developing more sustainable self-cleaning technologies. In addition, the broad spectrum of its physical antibacterial activity needs to be further verified, and its structural activity relationship is not comprehensive enough. A single antibacterial mechanism seems to be far from enough for one bactericidal or antiadhesion surface, but it should not prevent further research in this field. Therefore, the preparation of multifunctional composites based on physicochemical antibacterial micro-/nano-structures will be a great challenge. However, once this material can be prepared, its market prospect will be very large.

## Figures and Tables

**Table 1:** Basic information on different organisms inspiring scholars.

Biological Prototype	Strategy	Inspiration	Manufacture	Ref.
Mussel	Adhesion proteins; Surface morphology	A “flowering tree” structure on mussel shells; Micro-/nano-structures	Bionic Micro-/nano-structures	[31,32]
Coral	Natural antifoulants; Tentacle structure	Antifouling properties and harmonic antifouling surfaces	Simulate the structure and antifoulants of soft coral	[30,33]
Lotus	Cassie–Baxter state superhydrophobicity	Lotus effect	Lotus leaf-like hierarchical TiO_2_ structures	[19]
Gecko	Surface micro-/nano-structure	Self-cleaning and antifouling characters of skins and feet	Simulate Gecko’s feet-like micro-/nano-brush dual-structural surfaces	[18,34]
Shark	Surface micro-/nano-structure	Superhydrophobic/antifouling structures	Biomimetic shark skin surface	[28,29,35]

## Data Availability

No new data were created or analyzed in this study. Data sharing is not applicable to this article.

## References

[1] Harper R.A., Carpenter G.H., Proctor G.B., Harvey R.D., Gambogi R.J., Geonnotti A.R., Hider R., Jones S.A. (2019). Diminishing biofilm resistance to antimicrobial nanomaterials through electrolyte screening of electrostatic interactions. Colloids Surfaces B.

[2] Dieltjens L., Appermans K., Lissens M., Lories B., Kim W., Van der Eycken E.V., Foster K.R., Steenackers H.P. (2020). Inhibiting bacterial cooperation is an evolutionarily robust anti-biofilm strategy. Nat. Commun..

[3] Tang Y., Xu H., Wang X., Dong S., Guo L., Zhang S., Yang C., Liu X., Jiang X., Kan M. (2023). Advances in preparation and application of antibacterial hydrogels. J. Nanobiotechnol..

[4] Alotaibi G.F., Mamdouh A.B. (2021). Factors influencing bacterial biofilm formation and development. Am. J. Biomed. Sci. Res..

[5] Jiang J., Lv X., Cheng H., Yang D., Xu W., Hu Y., Song Y., Zeng G. (2024). Type I Photodynamic Antimicrobial Therapy: Principles, Progress, and Future Perspectives. Acta Biomater..

[6] Muhammad M.H., Idris A.L., Fan X., Guo Y., Yu Y., Jin X., Qiu J., Guan X., Huang T. (2020). Beyond risk: Bacterial biofilms and their regulating approaches. Front. Microbiol..

[7] Thomas R.E., Bennett C.T. (2021). Reducing biofilm infections in burn patients’ wounds and biofilms on surfaces in hospitals, medical facilities and medical equipment to improve burn care: A systematic review. Int. J. Environ. Res. Public Health.

[8] Jamal M., Ahmad W., Andleeb S., Jalil F., Imran M., Nawaz M.A., Hussain T., Ali M., Rafiq M., Kamil M.A. (2018). Bacterial biofilm and associated infections. J. Chin. Med. Assoc..

[9] Zafer M.M., Mohamed G.A., Ibrahim S.R.M., Ghosh S., Bornman C., Elfaky M.A. (2024). Biofilm-mediated infections by multidrug-resistant microbes: A comprehensive exploration and forward perspectives. Arch. Microbiol..

[10] Ali A., Zahra A., Kamthan M., Husain F.M., Albalawi T., Zubair M., Alatawy R., Abid M., Noorani M.S. (2023). Microbial biofilms: Applications, clinical consequences, and alternative therapies. Microorganisms.

[11] Yuan Z., Lin C., He Y., Tao B., Chen M., Zhang J., Liu P., Cai K. (2020). Near-Infrared Light-Triggered Nitric-Oxide-Enhanced Photodynamic Therapy and Low-Temperature Photothermal Therapy for Biofilm Elimination. ACS Nano.

[12] Elbourne A., Cheeseman S., Atkin P., Truong N.P., Syed N., Zavabeti A., Mohiuddin M., Esrafilzadeh D., Cozzolino D., McConville C.F. (2020). Antibacterial Liquid Metals: Biofilm Treatment via Magnetic Activation. ACS Nano.

[13] Koch K., Bhushan B., Barthlott W.J.P.I.M.S. (2009). Multifunctional surface structures of plants: An inspiration for biomimetics. Pro. Mater. Sci..

[14] Gorb E.V., Stanislav N.G. (2023). Petals Reduce Attachment of Insect Pollinators: A Case Study of the Plant Dahlia pinnata and the Fly Eristalis tenax. Insects..

[15] Hischen F., Reiswich V., Kupsch D., De Mecquenem N., Riedel M., Himmelsbach M., Weth A., Heiss E., Armbruster O., Heitz J. (2017). Adaptive camouflage: What can be learned from the wetting behaviour of the tropical flat bugs *Dysodius lunatus* and *Dysodius magnus*. Biol. Open.

[16] Loewenstein W.R. (1975). Camouflage by Integumentary Wetting in Bark Bugs. Science.

[17] Watson G.S., Green D.W., Schwarzkopf L., Li X., Cribb B.W., Myhra S., Watson J.A. (2015). A gecko skin micro/nano structure—A low adhesion, superhydrophobic, anti-wetting, self-cleaning, biocompatible, antibacterial surface. Acta Biomater..

[18] Li X., Cheung G.S., Watson G.S., Watson J.A., Green D.W. (2016). The nanotipped hairs of gecko skin and biotemplated replicas impair and/or kill pathogenic bacteria with high efficiency. Nanoscale.

[19] Liang Y., Yang E., Kim M., Kim S., Kim H., Byun J., Yanar N., Choi H. (2023). Lotus leaf-like SiO_2_ nanofiber coating on polyvinylidene fluoride nanofiber membrane for water-in-oil emulsion separation and antifouling enhancement. Chem. Eng. J..

[20] Lv Y., Song C., Hou Y., Shi M., Li Q., Zhang T. (2020). Bioinspired like lotus leaf hierarchical micropapillae structure for efficient oil-water separation and antibacterial performance. J. Disper. Sci. Technol..

[21] Li D., Lin Z., Zhu J., Yu J., Liu Z., Chen R., Liu Q., Liu P., Wang J. (2021). An engineering-oriented approach to construct rough micro/nano-structures for anticorrosion and antifouling application. Colloid Surface A.

[22] Andersson D.I., Hughes D. (2010). Antibiotic resistance and its cost: Is it possible to reverse resistance?. Nat. Rev. Microbiol..

[23] Davies S.C., Fowler T., Watson J., Livermore D.M., Walker D. (2013). Annual Report of the Chief Medical Officer: Infection and the rise of antimicrobial resistance. Lancet.

[24] Goyal P.K., Semwal A., Prakash A., Medhi B. (2019). Emerging antimicrobial resistance and newer tools to address the resistance. Indian J. Pharmacol..

[25] Cai Y., Bing W., Xu X., Zhang Y., Chen Z., Gu Z. (2021). Topographical nanostructures for physical sterilization. Drug Deliv. Transl. Res..

[26] Pogodin S., Hasan J., Baulin V.A., Webb H.K., Truong V.K., Phong Nguyen T.H., Boshkovikj V., Fluke C.J., Watson G.S., Watson J.A. (2013). Biophysical model of bacterial cell interactions with nanopatterned cicada wing surfaces. Biophys. J..

[27] Bhadra C.M., Khanh Truong V., Pham V.T., Al Kobaisi M., Seniutinas G., Wang J.Y., Juodkazis S., Crawford R.J., Ivanova E.P. (2015). Antibacterial titanium nano-patterned arrays inspired by dragonfly wings. Sci. Rep..

[28] Rostami S., Tekkeşin A.I., Ercan U.K., Garipcan B. (2022). Biomimetic sharkskin surfaces with antibacterial, cytocompatible, and drug delivery properties. Biomater. Adv..

[29] Tian G., Fan D., Feng X., Zhou H.J.R.A. (2021). Thriving artificial underwater drag-reduction materials inspired from aquatic animals: Progresses and challenges. RSC Adv..

[30] Bing W., Jin E., Tian L., Jin H., Liu Z. (2022). Construction and application of bionic antifouling coatings inspired by soft coral. Biosurface Biotribology.

[31] Guan Y., Chen R., Sun G., Liu Q., Liu J., Yu J., Lin C., Duan J., Wang J. (2021). The mussel-inspired micro-nano structure for antifouling: A flowering tree. J. Colloid Interface Sci..

[32] Li B., Tian T., Zhang X., Han C., Yun Y., Zhu X., Wu J. (2023). Mussels-inspired design a multi-level micro/nano re-entrant structure amphiphobic PVDF membrane with robust anti-fouling for direct contact membrane distillation. Desalination.

[33] Tian L., Jin E., Yu B., Sun H., Shang Y., Bing W. (2020). Novel anti-fouling strategies of live and dead soft corals (*Sarcophyton trocheliophorum*): Combined physical and chemical mechanisms. J. Bionic Eng..

[34] Du T., Ma S., Pei X., Wang S., Zhou F. (2017). Bio-inspired design and fabrication of micro/nano-brush dual structural surfaces for switchable oil adhesion and antifouling. Small.

[35] Pan L.C., Hsieh S.Y., Chen W.C., Lin F.T., Lu C.H., Cheng Y.L., Chien H.-W., Yang H. (2023). Self-Assembly of Shark Scale-Patterned Tunable Superhydrophobic/Antifouling Structures with Visual Color Response. ACS Appl. Mater. Interfaces..

[36] Cao Y., Jana S., Bowen L., Tan X., Liu H., Rostami N., Brown J., Jakubovics N.S., Chen J. (2019). Hierarchical Rose Petal Surfaces Delay the Early-Stage Bacterial Biofilm Growth. Langmuir.

[37] Oopath S.V., Baji A., Abtahi M., Luu T.Q., Vasilev K., Truong V.K. (2023). Nature-Inspired Biomimetic Surfaces for Controlling Bacterial Attachment and Biofilm Development. Adv. Mater. Interface..

[38] Jia B., Du X., Wang W., Qu Y., Liu X., Zhao M., Li W., Li Y.-Q. (2022). Nanophysical antimicrobial strategies: A rational deployment of nanomaterials and physical stimulations in combating bacterial infections. Adv. Sci..

[39] Yuan Y., Zhang Y. (2017). Enhanced biomimic bactericidal surfaces by coating with positively-charged ZIF nano-dagger arrays. Nanomed. Nanotechnol. Biol. Med..

[40] Hochbaum A.I., Aizenberg J. (2010). Bacteria pattern spontaneously on periodic nanostructure arrays. Nano Lett..

[41] Xu L.-C., Christopher A.S. (2022). Submicron topography design for controlling staphylococcal bacterial adhesion and biofilm formation. J. Biomed. Mater. Res. A.

[42] Kargar M., Pruden A., Ducker W.A. (2014). Preventing bacterial colonization using colloidal crystals. J. Mater. Chem. B.

[43] Pingle H., Wang P.Y., Thissen H., Kingshott P. (2018). Controlled Attachment of Pseudomonas aeruginosa with Binary Colloidal Crystal-Based Topographies. Small.

[44] Sui Z., Wang J., Wu C., Niu J., Zhu J., Zhou L. (2023). Research on the surface characterization, corrosion and bioactivity of nano-featured tantalum coating on selective electron beam melted Ti6Al4V alloy. J. Alloy. Compd..

[45] Felipe V., Ivan N.-B., Alejandra J.-M., Jaime H.J., Martal B.-E. (2018). Nano-engineering safer-by-design nanoparticle based moth-eye mimetic bactericidal and cytocompatible polymer surfaces. RSC Adv..

[46] Michalska M., Gambacorta F., Divan R., Aranson I.S., Sokolov A., Noirot P., Laible P.D. (2018). Tuning antimicrobial properties of biomimetic nanopatterned surfaces. Nanoscale.

[47] Yao L., Wang H., Li L., Cao Z., Dong Y., Yao L., Lou W., Zheng S., Shi Y., Shen X. (2022). Development and evaluation of osteogenesis and antibacterial properties of strontium/silver-functionalized hierarchical micro/nano-titanium implants. Mater. Design.

[48] Tsui K.H., Li X., Tsoi J.K.H., Leung S.F., Lei T., Chak W.Y., Zhang C., Chen J., Cheung G.S.P., Fan Z. (2018). Low-cost, flexible, disinfectant-free and regular-array three-dimensional nanopyramid antibacterial films for clinical applications. Nanoscale.

[49] Ganjian M., Modaresifar K., Ligeon M.R., Kunkels L.B., Tümer N., Angeloni L., Hagen C.W., Otten L.G., Hagedoorn P.L., Apachitei I. (2019). Nature helps: Toward bioinspired bactericidal nanopatterns. Adv. Mater. Interfaces.

[50] Yang X.M., Hou J.W., Tian Y., Zhao J., Sun Q., Zhou S. (2022). Antibacterial surfaces: Strategies and applications. Sci. China Technol. Sci..

[51] Riduan S.N., Zhang Y. (2021). Nanostructured surfaces with multimodal antimicrobial action. Acc. Chem. Res..

[52] Cai S., Wu C., Yang W., Liang W., Yu H., Liu L. (2020). Recent advance in surface modification for regulating cell adhesion and behaviors. Nanotechnol. Rev..

[53] Stratakis E., Bonse J., Heitz J., Siegel J., Tsibidis G.D., Skoulas E., Papadopoulos A., Mimidis A., Joel A.C., Comanns P. (2020). Laser engineering of biomimetic surfaces. Mater. Sci. Eng. R Rep..

[54] Thwaites J.J., Surana U.C. (1991). Mechanical properties of Bacillus subtilis cell walls: Effects of removing residual culture medium. J. Bacteriol..

[55] Chen Y., Ao J., Zhang J., Gao J., Hao L., Jiang R., Zhang Z., Liu Z., Zhao J., Ren L. (2023). Bioinspired superhydrophobic surfaces, inhibiting or promoting microbial contamination?. Mater. Today.

[56] Xie X., Xu A.M., Angle M.R., Tayebi N., Verma P., Melosh N.A. (2013). Mechanical model of vertical nanowire cell penetration. Nano Lett..

[57] Mu M., Liu S., DeFlorio W., Hao L., Wang X., Salazar K.S., Taylor M., Castillo A., Cisneros-Zevallos L., Oh J.K. (2023). Influence of surface roughness, nanostructure, and wetting on bacterial adhesion. Langmuir.

[58] Chopra D., Gulati K., Ivanovski S. (2021). Bed of nails: Bioinspired nano-texturing towards antibacterial and bioactivity functions. Mater. Today Adv..

[59] Bandara C.D., Singh S., Afara I.O., Wolff A., Tesfamichael T., Ostrikov K., Oloyede A. (2017). Bactericidal Effects of Natural Nanotopography of Dragonfly Wing on *Escherichia coli*. ACS Appl. Mater. Interfaces.

[60] Linklater D.P., Juodkazis S., Rubanov S., Ivanova E.P. (2017). Comment on Bactericidal Effects of Natural Nanotopography of Dragonfly Wing on *Escherichia coli*. ACS Appl. Mater. Interfaces.

[61] Jiang S., Lin K., Cai M. (2020). ZnO nanomaterials: Current advancements in antibacterial mechanisms and applications. Front. Chem..

[62] Mujeeb A.A., Khan N.A., Jamal F., Badre Alam K.F., Saeed H., Kazmi S., Alshameri A.W.F., Kashif M., Ghazi I., Owais M. (2020). *Olax Scandens* Mediated Biogenic Synthesis of Ag-Cu Nanocomposites: Potential Against Inhibition of Drug-Resistant Microbes. Front. Chem..

[63] Franco D., Calabrese G., Guglielmino S.P.P., Conoci S. (2022). Metal-based nanoparticles: Antibacterial mechanisms and biomedical application. Microorganisms.

[64] Wang Y., Yang Y., Shi Y., Song H., Yu C. (2020). Antibiotic-free antibacterial strategies enabled by nanomaterials: Progress and perspectives. Adv. Mater..

[65] Aldeen T.S., Mohamed H.E.A., Maaza M. (2022). ZnO nanoparticles prepared via a green synthesis approach: Physical properties, photocatalytic and antibacterial activity. J. Phys. Chem. Solids.

[66] Gao B., Fu J., Huo K., Zhang W., Xie Y., Chu P.K. (2011). Quasi-Aligned Ag–Nb_2_O_5_ Nanobelt Arrays with Enhanced Photocatalytic and Antibacterial Activities. J. Am. Ceram. Soc..

[67] Tang Y., Sun H., Qin Z., Yin S., Tian L., Liu Z. (2020). Bioinspired photocatalytic ZnO/Au nanopillar-modified surface for enhanced antibacterial and antiadhesive property. Chem. Eng. J..

